# 3-(2,4-Dichloro­phen­yl)-5-methyl-1,2,4-oxadiazole

**DOI:** 10.1107/S1600536810007932

**Published:** 2010-03-06

**Authors:** Hoong-Kun Fun, Mohd Mustaqim Rosli, Sankappa Rai, Arun M Isloor, Prakash Shetty

**Affiliations:** a X-ray Crystallography Unit, School of Physics, Universiti Sains Malaysia, 11800 USM, Penang, Malaysia; b Syngene International Ltd, Biocon Park, Plot Nos 2 & 3, Bommasandra 4^th^ Phase, Jigani Link Rd, Bangalore 560100, India; c Department of Chemistry, Organic Chemistry Division, National Institute of Technology-Karnataka, Surathkal, Mangalore 575 025, India; d Department of Printing, Manipal Institute of Technology, Manipal 576 104, India

## Abstract

In the title compound, C_9_H_6_Cl_2_N_2_O, the dihedral angle between the oxadiazole and benzene rings is 1.7 (2)°. In the crystal, the mol­ecules are linked into chains along the *b* axis by short inter­molecular Cl⋯O contacts [3.019 (3) Å].

## Related literature

For general background and the biological activity of oxa­diazole compounds, see: Andersen *et al.* (1994 ▶); Clitherow *et al.* (1996 ▶); Showell *et al.* (1991 ▶); Swain *et al.* (1991 ▶); Watjen *et al.* (1989 ▶). For a related structure, see: Wang *et al.* (2006 ▶). For the stability of the temperature controller used for the data collection, see: Cosier & Glazer (1986 ▶).
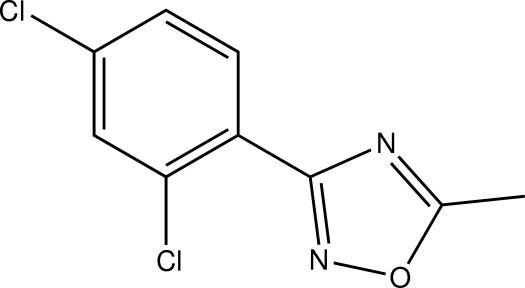


## Experimental

### 

#### Crystal data

C_9_H_6_Cl_2_N_2_O
*M* *_r_* = 229.06
Monoclinic, 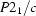

*a* = 3.8252 (7) Å
*b* = 21.678 (4) Å
*c* = 11.0833 (19) Å
β = 92.421 (4)°
*V* = 918.3 (3) Å^3^
*Z* = 4
Mo *K*α radiation
μ = 0.67 mm^−1^
*T* = 100 K
0.28 × 0.17 × 0.11 mm

#### Data collection

Bruker SMART APEXII CCD area-detector diffractometer
Absorption correction: multi-scan (*SADABS*; Bruker, 2005 ▶) *T*_min_ = 0.833, *T*_max_ = 0.929
7920 measured reflections
2076 independent reflections
1709 reflections with *I* > 2σ(*I*)
*R*_int_ = 0.048

#### Refinement

*R*[*F* ^2^ > 2σ(*F* ^2^)] = 0.056
*wR*(*F* ^2^) = 0.155
*S* = 1.20
2076 reflections
128 parameters
H-atom parameters constrained
Δρ_max_ = 0.72 e Å^−3^
Δρ_min_ = −0.56 e Å^−3^

### 

Data collection: *APEX2* (Bruker, 2005 ▶); cell refinement: *SAINT* (Bruker, 2005 ▶); data reduction: *SAINT*; program(s) used to solve structure: *SHELXTL* (Sheldrick, 2008 ▶); program(s) used to refine structure: *SHELXTL*; molecular graphics: *SHELXTL*; software used to prepare material for publication: *SHELXTL* and *PLATON* (Spek, 2009 ▶).

## Supplementary Material

Crystal structure: contains datablocks global, I. DOI: 10.1107/S1600536810007932/ci5045sup1.cif

Structure factors: contains datablocks I. DOI: 10.1107/S1600536810007932/ci5045Isup2.hkl

Additional supplementary materials:  crystallographic information; 3D view; checkCIF report

## Figures and Tables

**Fig. 1.**
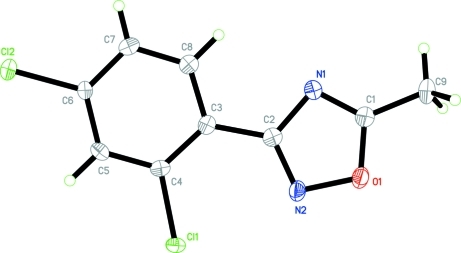
The molecular structure of the title compound, showing 50% probability displacement ellipsoids and the atom-numbering scheme.

**Fig. 2.**
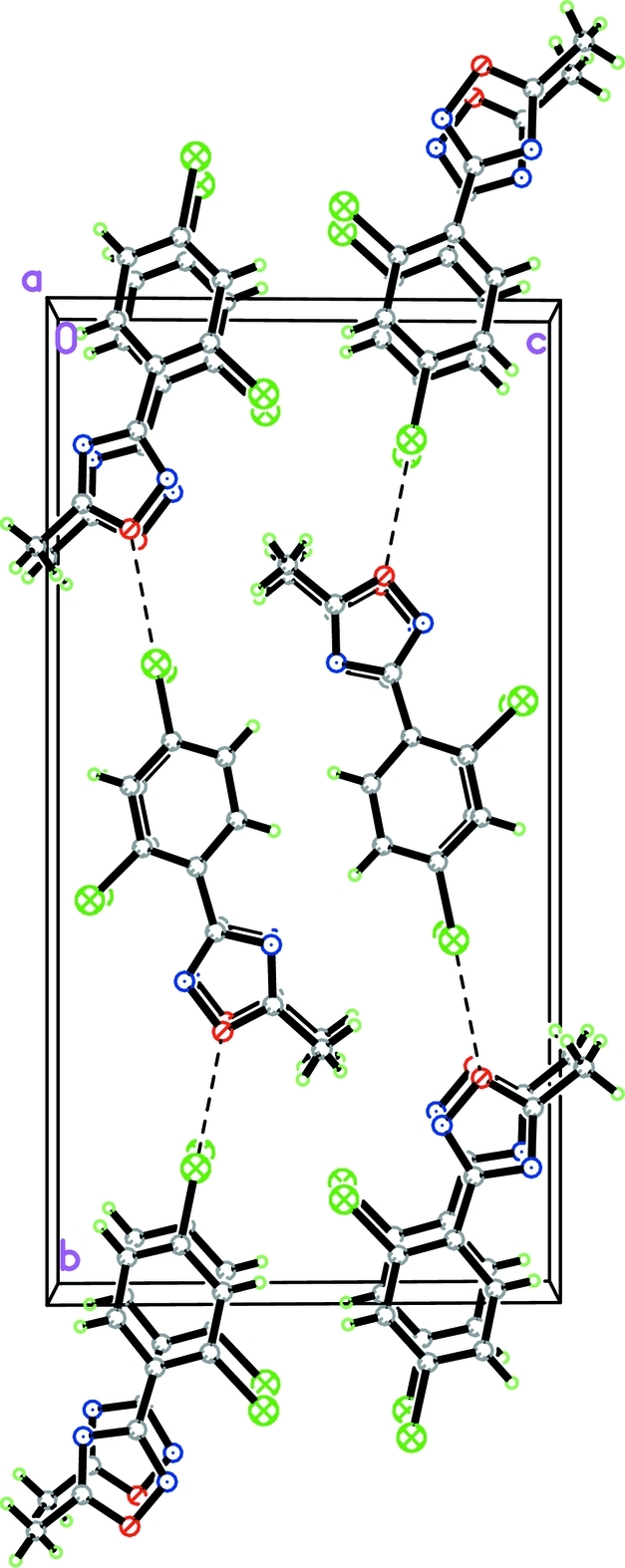
The crystal structure of the title compound, showing chains along the b axis.

## supplementary crystallographic information

### Comment

Heterocyclic compounds are important in recent years due to pharmacological activities. Nitrogen, oxygen containing five- and six-membered heterocyclic compounds have enormous significance in the field of medicinal chemistry. Oxadiazoles play a very vital role in the preparation of various biologically active drugs with anti-inflammatory (Andersen *et al.*,  1994), anti-cancer (Showell *et al.*,  1991), anti-HIV (Watjen *et al.*, 1989), anti-diabetic and anti-microbial (Swain *et al.*,  1991) properties. The results of biological studies showed that oxadiazole derivatives are molecules with maximum anti-inflammatory, analgesic and minimum ulcerogenic and lipid per-oxidation (Clitherow *et al.*,  1996) properties.

Bond lengths and angles are normal (Wang *et al.*, 2006). The mean plane of the oxadiazole ring (C1/C2/N1/N2/O1) is almost coplanar with the mean plane of the C3–C8 benzene ring (Fig. 1), with a dihedral angle of 1.7 (2)°.

The molecules are linked by Cl2···O1(-x, 1/2+y, 3/2-z) short contacts [3.019 (3) Å;] to form chains along the *b* axis (Fig.2).

### Experimental

The title compound was prepared by heating a solution of 2,4-dichloro-N'-hydroxy-benzamidine (1 g, 0.0042 mol) and acetyl chloride (0.38 g, 0.004 mol) in pyridine (30 ml) at 387 K for 1.5 h and the contents were concentrated under vacuum. Further purification was done by column chromatography. The solid obtained was recrystallized using dichloromethane (yield: 1.0 g (76%); m.p. 371-372 K).

### Refinement

H atoms were placed in calculated positions [C–H = 0.93–0.96 Å] and refined as riding with U_iso_(H) = 1.2_eq_(C) or 1.5U_eq_(methyl C). A rotating group model was used for the methyl group.

### Crystal data

**Table:**

C_9_H_6_Cl_2_N_2_O	*F*(000) = 464
*M**_r_* = 229.06	*D*_x_ = 1.657 Mg m^−^^3^
Monoclinic, *P*2_1_/*c*	Mo *K*α radiation, λ = 0.71073 Å
Hall symbol: -P 2ybc	Cell parameters from 3719 reflections
*a* = 3.8252 (7) Å	θ = 2.6–31.1°
*b* = 21.678 (4) Å	µ = 0.67 mm^−^^1^
*c* = 11.0833 (19) Å	*T* = 100 K
β = 92.421 (4)°	Block, colourless
*V* = 918.3 (3) Å^3^	0.28 × 0.17 × 0.11 mm
*Z* = 4	

### Data collection

**Table:**

Bruker SMART APEXII CCD area-detector diffractometer	2076 independent reflections
Radiation source: fine-focus sealed tube	1709 reflections with *I* > 2σ(*I*)
graphite	*R*_int_ = 0.048
φ and ω scans	θ_max_ = 27.5°, θ_min_ = 1.9°
Absorption correction: multi-scan (*SADABS*; Bruker, 2005)	*h* = −4→4
*T*_min_ = 0.833, *T*_max_ = 0.929	*k* = −26→28
7920 measured reflections	*l* = −13→14

### Refinement

**Table:**

Refinement on *F*^2^	Primary atom site location: structure-invariant direct methods
Least-squares matrix: full	Secondary atom site location: difference Fourier map
*R*[*F*^2^ > 2σ(*F*^2^)] = 0.056	Hydrogen site location: inferred from neighbouring sites
*wR*(*F*^2^) = 0.155	H-atom parameters constrained
*S* = 1.20	*w* = 1/[σ^2^(*F*_o_^2^) + (0.0428*P*)^2^ + 4.0517*P*] where *P* = (*F*_o_^2^ + 2*F*_c_^2^)/3
2076 reflections	(Δ/σ)_max_ = 0.001
128 parameters	Δρ_max_ = 0.72 e Å^−^^3^
0 restraints	Δρ_min_ = −0.56 e Å^−^^3^

### Special details

**Table:**

Experimental. The crystal was placed in the cold stream of an Oxford Cyrosystems Cobra open-flow nitrogen cryostat (Cosier & Glazer, 1986) operating at 100.0 (1) K.
Geometry. All esds (except the esd in the dihedral angle between two l.s. planes) are estimated using the full covariance matrix. The cell esds are taken into account individually in the estimation of esds in distances, angles and torsion angles; correlations between esds in cell parameters are only used when they are defined by crystal symmetry. An approximate (isotropic) treatment of cell esds is used for estimating esds involving l.s. planes.
Refinement. Refinement of F^2^ against ALL reflections. The weighted R-factor wR and goodness of fit S are based on F^2^, conventional R-factors R are based on F, with F set to zero for negative F^2^. The threshold expression of F^2^ > 2sigma(F^2^) is used only for calculating R-factors(gt) etc. and is not relevant to the choice of reflections for refinement. R-factors based on F^2^ are statistically about twice as large as those based on F, and R- factors based on ALL data will be even larger.

### Fractional atomic coordinates and isotropic or equivalent isotropic displacement parameters (Å^2^)

**Table:**

	*x*	*y*	*z*	*U*_iso_*/*U*_eq_	
Cl1	0.2530 (2)	0.40084 (4)	0.92173 (8)	0.0163 (2)	
Cl2	0.1238 (3)	0.63730 (4)	0.78985 (9)	0.0185 (3)	
O1	−0.2054 (8)	0.27327 (13)	0.6574 (3)	0.0220 (7)	
N1	−0.3467 (8)	0.35961 (15)	0.5636 (3)	0.0158 (7)	
N2	−0.0709 (10)	0.32171 (16)	0.7322 (3)	0.0194 (7)	
C1	−0.3637 (11)	0.30045 (18)	0.5605 (4)	0.0173 (8)	
C2	−0.1651 (10)	0.37119 (18)	0.6715 (3)	0.0140 (8)	
C3	−0.0869 (9)	0.43519 (17)	0.7088 (3)	0.0124 (7)	
C4	0.0928 (10)	0.45392 (18)	0.8165 (3)	0.0137 (8)	
C5	0.1523 (9)	0.51553 (18)	0.8418 (4)	0.0136 (7)	
H5A	0.2684	0.5272	0.9136	0.016*	
C6	0.0369 (10)	0.56000 (17)	0.7588 (3)	0.0127 (7)	
C7	−0.1429 (10)	0.54398 (18)	0.6533 (4)	0.0151 (8)	
H7A	−0.2240	0.5740	0.5990	0.018*	
C8	−0.1993 (10)	0.48214 (18)	0.6303 (4)	0.0153 (8)	
H8A	−0.3186	0.4712	0.5586	0.018*	
C9	−0.5226 (12)	0.2592 (2)	0.4666 (4)	0.0237 (9)	
H9A	−0.6428	0.2835	0.4055	0.036*	
H9B	−0.6856	0.2319	0.5028	0.036*	
H9C	−0.3422	0.2354	0.4308	0.036*	

### Atomic displacement parameters (Å^2^)

**Table:**

	*U*^11^	*U*^22^	*U*^33^	*U*^12^	*U*^13^	*U*^23^
Cl1	0.0168 (5)	0.0182 (5)	0.0138 (5)	0.0028 (3)	−0.0011 (3)	0.0023 (4)
Cl2	0.0197 (5)	0.0140 (4)	0.0219 (5)	−0.0024 (3)	0.0025 (4)	−0.0017 (4)
O1	0.0309 (17)	0.0139 (14)	0.0213 (15)	−0.0017 (12)	0.0017 (12)	−0.0018 (12)
N1	0.0130 (15)	0.0175 (16)	0.0172 (17)	0.0000 (12)	0.0018 (12)	−0.0022 (13)
N2	0.0257 (19)	0.0147 (16)	0.0178 (18)	−0.0002 (14)	0.0018 (14)	−0.0032 (13)
C1	0.0148 (19)	0.0194 (19)	0.018 (2)	0.0000 (15)	0.0051 (15)	−0.0007 (15)
C2	0.0103 (17)	0.0185 (19)	0.0138 (18)	0.0022 (14)	0.0081 (14)	0.0000 (14)
C3	0.0078 (17)	0.0152 (18)	0.0150 (18)	0.0009 (13)	0.0080 (14)	−0.0011 (14)
C4	0.0126 (18)	0.0171 (19)	0.0119 (19)	0.0043 (13)	0.0054 (14)	0.0016 (14)
C5	0.0070 (16)	0.0208 (19)	0.0132 (18)	0.0017 (14)	0.0022 (13)	−0.0021 (15)
C6	0.0106 (17)	0.0134 (17)	0.0144 (18)	−0.0008 (13)	0.0033 (13)	−0.0027 (14)
C7	0.0106 (18)	0.0174 (19)	0.0177 (19)	0.0033 (14)	0.0056 (14)	0.0033 (15)
C8	0.0114 (17)	0.0181 (19)	0.017 (2)	0.0005 (14)	0.0055 (14)	0.0004 (15)
C9	0.025 (2)	0.018 (2)	0.029 (2)	−0.0030 (16)	0.0041 (18)	−0.0072 (17)

### Geometric parameters (Å, °)

**Table:**

Cl1—C4	1.732 (4)	C4—C5	1.382 (5)
Cl2—C6	1.740 (4)	C5—C6	1.391 (5)
O1—C1	1.346 (5)	C5—H5A	0.93
O1—N2	1.421 (4)	C6—C7	1.376 (5)
N1—C1	1.285 (5)	C7—C8	1.380 (6)
N1—C2	1.381 (5)	C7—H7A	0.93
N2—C2	1.308 (5)	C8—H8A	0.93
C1—C9	1.483 (6)	C9—H9A	0.96
C2—C3	1.475 (5)	C9—H9B	0.96
C3—C8	1.396 (5)	C9—H9C	0.96
C3—C4	1.411 (5)		
			
C1—O1—N2	106.4 (3)	C6—C5—H5A	120.3
C1—N1—C2	103.2 (3)	C7—C6—C5	121.3 (4)
C2—N2—O1	102.8 (3)	C7—C6—Cl2	119.7 (3)
N1—C1—O1	113.3 (4)	C5—C6—Cl2	119.0 (3)
N1—C1—C9	129.8 (4)	C6—C7—C8	118.1 (4)
O1—C1—C9	116.9 (4)	C6—C7—H7A	121.0
N2—C2—N1	114.4 (4)	C8—C7—H7A	121.0
N2—C2—C3	125.4 (4)	C7—C8—C3	123.5 (4)
N1—C2—C3	120.2 (3)	C7—C8—H8A	118.3
C8—C3—C4	116.4 (3)	C3—C8—H8A	118.3
C8—C3—C2	117.2 (3)	C1—C9—H9A	109.5
C4—C3—C2	126.4 (3)	C1—C9—H9B	109.5
C5—C4—C3	121.3 (3)	H9A—C9—H9B	109.5
C5—C4—Cl1	117.1 (3)	C1—C9—H9C	109.5
C3—C4—Cl1	121.6 (3)	H9A—C9—H9C	109.5
C4—C5—C6	119.4 (3)	H9B—C9—H9C	109.5
C4—C5—H5A	120.3		
			
C1—O1—N2—C2	0.1 (4)	C8—C3—C4—C5	0.0 (5)
C2—N1—C1—O1	−0.4 (5)	C2—C3—C4—C5	179.8 (3)
C2—N1—C1—C9	−179.1 (4)	C8—C3—C4—Cl1	−179.1 (3)
N2—O1—C1—N1	0.2 (5)	C2—C3—C4—Cl1	0.6 (5)
N2—O1—C1—C9	179.1 (3)	C3—C4—C5—C6	−0.8 (6)
O1—N2—C2—N1	−0.4 (4)	Cl1—C4—C5—C6	178.4 (3)
O1—N2—C2—C3	−179.3 (3)	C4—C5—C6—C7	1.6 (6)
C1—N1—C2—N2	0.5 (5)	C4—C5—C6—Cl2	−178.3 (3)
C1—N1—C2—C3	179.5 (3)	C5—C6—C7—C8	−1.5 (6)
N2—C2—C3—C8	178.0 (4)	Cl2—C6—C7—C8	178.4 (3)
N1—C2—C3—C8	−0.8 (5)	C6—C7—C8—C3	0.7 (6)
N2—C2—C3—C4	−1.8 (6)	C4—C3—C8—C7	0.1 (6)
N1—C2—C3—C4	179.4 (4)	C2—C3—C8—C7	−179.7 (4)
